# Prevalence, trend and risk factors for antiretroviral therapy discontinuation among HIV-infected adults in Ethiopia in 2003-2015

**DOI:** 10.1371/journal.pone.0179533

**Published:** 2017-06-16

**Authors:** Hailay Abrha Gesesew, Paul Ward, Kifle Woldemichael, Lillian Mwanri

**Affiliations:** 1 Public Health, Flinders University, Adelaide, Australia; 2 Epidemiology, Jimma University, Jimma, Ethiopia; Istituto di Genetica Molecolare, ITALY

## Abstract

**Background:**

It is well acknowledged that antiretroviral therapy (ART) discontinuation hampers the progress towards achieving the UNAIDS treatment targets that aim to treat 90% of HIV diagnosed patients and achieve viral suppression for 90% of those on treatment. Nevertheless, the magnitude, trend and risk factors for ART discontinuation have not been explored extensively. We carried out a retrospective data analysis to assess prevalence, trend and risk factors for ART discontinuation among adults in Southwest Ethiopia.

**Methods:**

12 years retrospective cohort analysis was performed with 4900 HIV-infected adult patients between 21 June 2003 and 15 March 2015 registered at the ART clinic at Jimma University Teaching Hospital. ART discontinuation could be loss to follow-up, defaulting and/or stopping medication while remaining in care. Because data for 2003 and 2015 were incomplete, the 10 years data were used to describe trends for ART discontinuation using a line graph. We used binary logistic regression to identify factors that were correlated with ART discontinuation. To handle missing data, we applied multiple imputations assuming missing at random pattern.

**Results:**

In total, 4900 adult patients enrolled on ART, of whom 1090 (22.3%) had discontinued, 954 (19.5%) had transferred out, 300 (6.1%) had died, 2517 (51.4%) were alive and on ART, and the remaining 39 (0.8%) had unknown outcome status. The trend of ART discontinuation showed an upward direction in the recent times and reached a peak, accounting for a magnitude of 10%, in 2004 and 2005. Being a female (AOR = 2.1, 95%CI: 1.7–2.8), having an immunological failure (AOR = 2.3, 1.9–8.2), having tuberculosis/HIV co-infection (AOR = 1.5, 1.1–2.1) and no previous history of HIV testing (AOR = 1.8, 1.4–2.9) were the risk factors for ART discontinuation.

**Conclusions:**

One out of five adults had discontinued from ART, and the trend of ART discontinuation increased recently. Discontinued adults were more likely to be females, tuberculosis/HIV co-infected, with immunological failure and no history of HIV testing. Therefore, it is vital to implement effective programs such as community ART distribution and linkage-case-management to enhance ART linkage and retention.

## Introduction

Globally, 38.8 million people were living with human immunodeficiency virus (HIV), 2.5 million new HIV infections, and 1.2 million HIV/AIDS (acquired immunodeficiency syndrome) deaths in 2015[1]. Sub-Saharan Africa (SSA) contributed 76% (29 million) of the total HIV-infected people, 76% (1.9 million) of the total new HIV infections, and 75% (0.9 million) of the total HIV/AIDS deaths[1]. The advent of antiretroviral therapy (ART) in 1996 significantly reduced HIV-related deaths[2]. The global ART coverage in 2015 was low[3] (40.6%), with North Africa and Middle East having the lowest coverage (19%). High-income countries had the highest coverage (67%), and SSA had coverage of about 42%[1]. In Ethiopia, there were 768,040 HIV-infected people, 39,140 new HIV infections, and 28,650 HIV/AIDS deaths in 2015[1]. The ART coverage in Ethiopia in 2015 was moderate[3] (52%)[1].

Optimum clinical and public health achievements of ART requires consistent long-term adherence[4]. Nevertheless, ART discontinuation—interruptions to ART due to loss to follow-up (LTFU), defaulting or total stoppage of the treatment—is a big challenge. Additionally, ART discontinuation (discontinuation) causes drug resistance[5], diminishes the immunological benefit of treatment [6, 7], and increases AIDS-related morbidity and mortality [5, 8]. Discontinuation has been recognized as an impediment for attainment of the second 90 of the Joint United Nations Program on HIV and AIDS (UNAIDS) 90-90-90 treatment targets (sustainable provision of treatment for 90% of patients diagnosed with HIV) as it affects the sustainable intake of the treatment. Furthermore, discontinuation affects the performance of the third 90 of the UNAIDS 90-90-90 that aimed at achieving 90% of the virological success of patients on ART. This is because ART interruption lowers the efficacy of the treatment and subsequently leads to diminishing the number of CD4 cells, increases the number of viral counts[5], and then to failing immunological or virological success. Uganda reported 84% of virological suppression due to strong retention[9].

The magnitude of discontinuation in 2009 and 2012 was between 9–34% in Asia[10, 11] and 13.7–57.4% in Africa[12, 13]. In Ethiopia, the prevalence of discontinuation in 2012 and 2014 was between 9.8–31.4%[14, 15]. Previous studies in Ethiopia reported that demographic, behavioral, clinical and institutional factors were reported to contribute for discontinuation [14–18]. For example, smearing positive pulmonary tuberculosis, male gender, CD4 count <200 cells/μL, ambulatory functional status, having a mental illness, having HIV-negative partner, fear of stigma and side effects were the risk factors associated with discontnuation[14–18]. However, the previous studies that assessed either the prevalence[14, 18] or factors[14, 16–23] for discontinuation were all from the northern part of the country except two[16, 19]. This was also confirmed by our meta-analysis that reported that there were inadequate researches about ART discontinuation despite the large number of discontinued patients, and all the previous studies were concentrated on the northern and western part of Ethiopia[24].

The two studies[16, 19] that were conducted in the west part of the nation were case control studies and assessed only factors contributing for defaulting, reflecting that the magnitude of the problem was not explored. LTFU, another major contributor for discontinuation was not assessed. Unlike the rest of Ethiopia, the Southwest region is composed of different population groups. A refugee camp located near Jimma city, which takes refugees from different east African countries, contributes a number of HIV-infected patients enrolled in the ART clinic in Jimma University Teaching Hospital (JUTH). Furthermore, Jimma is also near Gambella region (Southwest Ethiopia), a region known to have the highest prevalence rate (6.5%) of HIV in Ethiopia[25], and the JUTH caters for both Jimma and Gambella zones. Since the prevalence of HIV in Southwestern region is higher (6.5%) than in other parts of the nation (<2%), it is necessary to understand whether the high prevalence is associated with other than factors for discontinuation identified in similar studies in Ethiopia.

Given the above gaps, and the clinical and public health significance of discontinuation, we have assessed the prevalence, trend and risk factors for ART discontinuation among adults by using 12 years (2003–2015) ART clinic data from JUTH in southwest Ethiopia. It is imperative to comprehensively understand the magnitude of the discontinuation problem and its influential factors in order to contextualise interventions to retain HIV patients in in care and to contribute to the Nation’s second and third UNAIDS treatment targets.

## Methods

### Study design, setting and participants

A retrospective cohort study was performed using data from 21 June 2003 to 15 March 2015 from the ART clinic at JUTH, Jimma, Southwest Ethiopia. The details of the study setting have been described in elsewhere [26]. The treatment protocol for Ethiopia is implemented using World Health Organization (WHO) ART treatment guideline [27] and National Guidelines for Comprehensive HIV Prevention, Care and Treatment: Federal Democratic Republic of Ethiopia, MoH [28]. According to the current treatment guidelines, HIV infected adults are eligible to start ART if their CD4 cell count is ≤ 500 cells/mm3 irrespective of WHO clinical stage, their WHO clinical stage is 3 or 4 irrespective CD4 cell count, and they are pregnant, breast feeding women, sero-discordant couples or diagnosed with active tuberculosis irrespective CD4 cell count. For all HIV-infected children below 15 years of age, ART is recommended irrespective of WHO clinical stage and CD4 cell count. All HIV-infected adults aged ≥15 years enrolled in ART care in JUTH were the target population. If the recorded outcome were death, transferred out or unknown, participants would be excluded from this study.

### Data source and procedures

**Fig 1:**
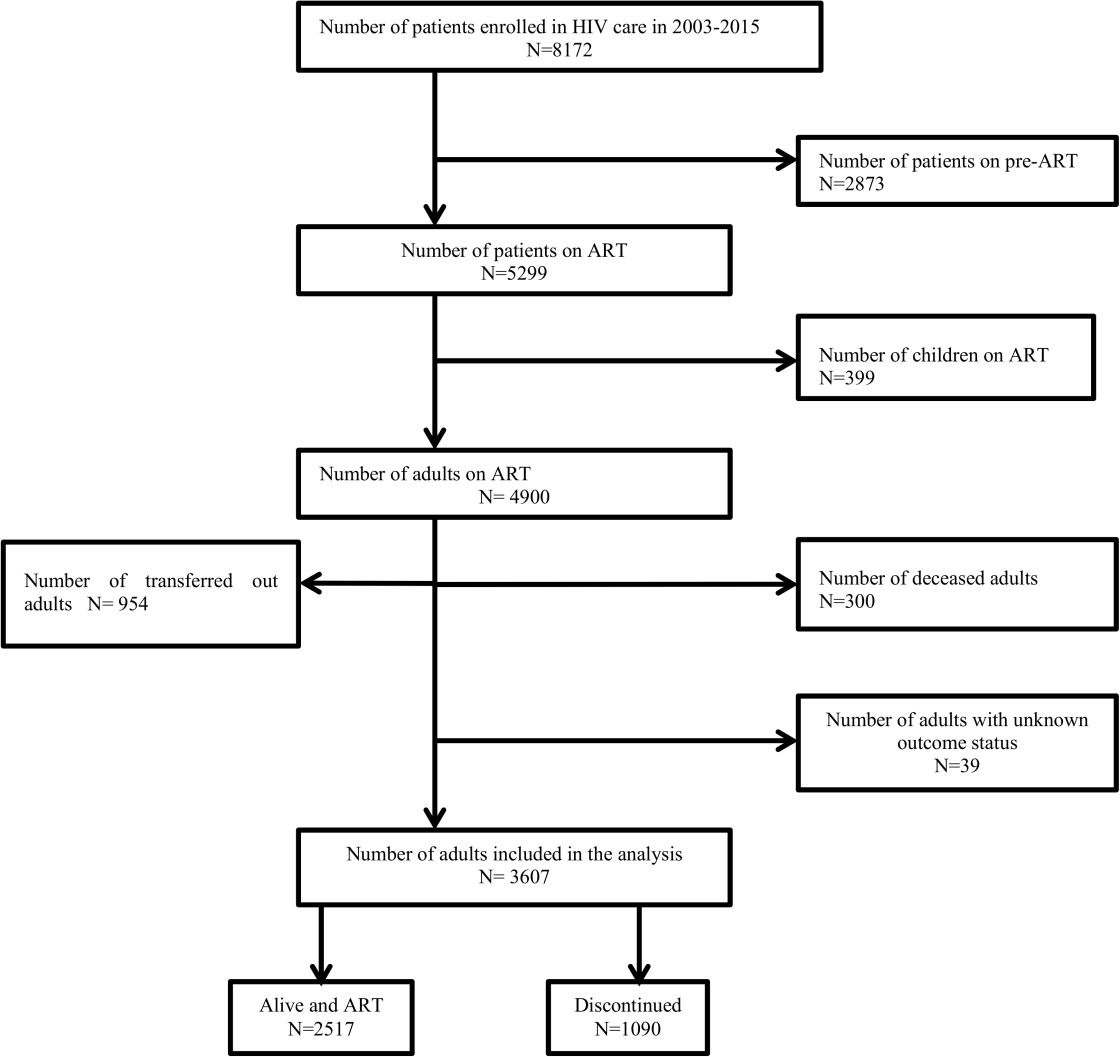
Schematic presentation of data extraction of ART discontinuation among HIV-infected adults in Jimma University Teaching Hospital, Southwest Ethiopia, 2003–2015. This figure presents the graphical demonstration of the data extraction process.

The data were extracted from JUTH electronic medical records (EMR) system called comprehensive care center patient application database (C-PAD). This system was designed since 2007, and data registered before 2007 were copied retrospectively to the electronic record. Fig 1 presents the schematic presentation of data extraction of discontinuation among HIV-infected adults in JUTH. In total, 4900 HIV-infected adults on ART were eligible or the study in the period between 2003 and 2015, and 3607 of them were included in the analysis. Clinicians record the clinical and non-clinical characteristics of the patients on paper, and afterward, data clerks entered the data into the EMR.

Two data clerks perform the data entry process, and this ensures the accuracy and reliability of the data. The International Center for AIDS Care and Support (ICAP) at Colombia University has been providing expertise assistance on the data management process and has also been providing checkups on completeness of the data. Weekly patient summary generated from the EMR system helps to flag patients with conditions that seek follow-up. Records would be excluded from a complete case analysis if outcome status of the person was death, transferred out or not recorded.

### Study variables and measurements

The dependent variable was discontinuation and was dichotomized as 1) alive and on ART, and 2) discontinued. Discontinuation refers to LTFU, defaulting and/or stopping medication while remaining in care. LTFU refers to patients who had been on ART treatment and had missed at least three clinical appointments but had not yet been classified as “dead” or “transferred out” (transferred). Defaulting refers to patients who had been on ART treatment and had missed less than three clinical appointments but had not yet been classified as “dead” or “transferred”. Stopping medication refers to patients who had stopped treatment due to any reason while they have remained in care. Transferred is an official transferring of patient to another ART clinic within or outside a catchment area.

The independent variables included age, sex, marital status, educational status, religion, ART adherence, cotrimoxazole adherence, clinical failure, immunological failure, treatment failure, late presentation for HIV care (LP), tuberculosis (Tb)/HIV co-infection, baseline functional status, history of HIV testing and ART shift. Educational status was categorized in to no education (could not read and write), primary (grade 1–8), and secondary and above (grades ≥9). Functional status was classified in to the following categories: i) work—able to perform usual work, ii) ambulatory—able to perform activity of daily living, and iii) bedridden—not able to perform activity of daily living. Table 1 reports the operational definitions of LP, adherence, clinical, immunological and treatment failures.

**Table 1:** Measurements for late presentation for HIV care, adherence, and immunological, clinical & treatment failures.

Late presentation for HIV care ^**a**^ [31–34]			
**Enrolled in 2003–11**		**Enrolled in 2012–15**	
CD4 lymphocyte count of <200 cells/ μl irrespective of WHO clinical stage at the time of first presentation to the HIV care		CD4 lymphocyte count of <350 cells/ μl irrespective of WHO clinical stage at the time of first presentation to the HIV care	
WHO clinical stage 3 or 4 irrespective of CD4 count at the time of first presentation to the HIV care ^b^		WHO clinical stage 3 or 4 irrespective of CD4 count at the time of first presentation to the HIV care	
**Adherence status** ^**c**^ [35]			
**Status**	**Percentage of prescribed ART intake**	**Number of missing doses out of 30**	**Number of missing doses out of 60**
Good	≥ 95%	<3	<4
Fair	85–95%	3–5	4–9
Poor	< 85	≥6	≥9
**Immunological and clinical failure** [36]			
**Clinical failure**		**Immunological failure**	**Treatment failure**
New clinical condition indicating severe immunodeficiency (with the exception of Tb, WHO clinical stage 4) after 6 months on ART		CD4 count falling to the baseline (or below) persistent CD4 levels below 100 cells/mm3 after or after 6 months	Having either clinical or immunological failures

ART: antiretroviral therapy; CD4: cluster for differentiation 4; HIV: human immunodeficiency virus; WHO: World Health Organization; Tb: Tuberculosis

^a^ The definition for late presentation for HIV care among Tb/HIV co-infected population was only based on the CD4 criteria.

^b^ **WHO clinical Stage 3** was defined if one of the following is present in an HIV diagnosed patient: weight loss of >10% body weight, chronic diarrhea for >1 month, fever for >1 month, oral candidiasis, oral hairy leukoplakia, or pulmonary Tb within the previous year, or severe bacterial infections; **WHO clinical Stage 4** was defined if one of the following is present in an HIV diagnosed patient: HIV wasting syndrome, PCP, toxoplasmosis of the brain, cryptosporidiosis or isosporiasis with diarrhea for >1 month, cytomegalovirus disease of an organ other than liver, spleen or lymph node, herpes simplex virus infection, progressive multifocal leukoencephalopathy, candidiasis, extra-pulmonary Tb, lymphoma, kaposi’s sarcoma, HIV encephalopathy

^c^ Clinicians and pharmacists ask patients and check the pill container to collect the number of missing doses or days

### Statistical analyses

Data cleaning and exploration were performed prior to analysis. Descriptive analysis was undertaken to explore the frequency tables and proportions for categorical data; and mean, median, range and line graph for continuous data. We described ten years trends for discontinuation (data for years 2003 and 2015 were excluded since the number of months was incomplete) using a line graph. The number of alive and on ART patients was calculated by subtracting the number of patients who dead, discontinued and Transferred from the total number of patients on the cohort. We checked for multicollinearity and potential interactions, and none was found.

We used binary logistic regression to identify factors that were correlated with discontinuation. To choose the candidate variables (P<0.25 was considered) to multiple logistic regression, we applied bivariate logistic regression analysis. P-value ≤ 0.05 was considered as a cutoff value for statistical significance in the final model. To handle missing data, we performed multiple imputations (n = 5) assuming missing at random (MAR) pattern[29], and we reported a model with pooled imputed values[30]. To check goodness of fit of the final model, we applied Hosmer and Lemeshow test and was found fit. To summarize the data, we reported odds ratio and 95% confidence interval. For all data analyses, we used Statistical Package for the Social Sciences (SPSS) version 22.0.

### Ethical statement

Ethical clearance was sought from Social and Behavioral Research Ethics Committee (SBREC) at Flinders University (Project number: 7086) and Institutional Review Board (IRB) of College of Health Sciences at Jimma University (Ref No: RPGC/386/2016). The data access permission was obtained from JUTH board, and the IRB waived the need for consent. No participant was involved in the study—we did simply extract anonymised data from the record.

## Results

### Characteristics of study participants

Of 8172 patients enrolled in HIV care program from 21 June 2003 to 15 March 2015, 4900 HIV-infected adults had documented commencement of ART (Fig 1). Table 2 demonstrates the characteristics of HIV patients on ART. Of the total, 80.3% were aged 25–50 years, 59.8% were females, 48.7% were married, 67.4% were Christians, and 39.1% completed primary education. The median CD4 count was 156 (0–1313) cells/mm3, and 54.3% of the participants had baseline WHO clinical stage 3 or 4. A total of 1367 (27.9%) patients were deemed Tb/HIV co-infection over the whole study period. The median time on ART was 49 months, and the estimated survival time was 121.9 (120.3–123.5) months.

**Table 2:** Clinical & non-clinical characteristics of HIV infected people enrolled on ART care in Southwest Ethiopia from 2003 to 2015.

Variable		N (%), N = 4900
Age in years	15-<25	711 (14.5)
	25-<50	3937 (80.3)
	50+	252 (5.2)
	Median (range) age in years	30 (15–81)
ART follow up time in months, median (range)		49 (0–137)
Estimated survival time in months, median (95%CI)		121.9 (120.3–123.5)
Sex	Male	1971 (40.2)
	Female	2929 (59.8)
Marital status ^b^	Never married	897 (20.9)
	Married	2094 (48.7)
	Separated or divorced	837 (19.4)
	Widowed	474 (11.0)
Education ^b^	No education	945 (21.9)
	Primary	1687 (39.1)
	Secondary and above	1685 (39)
Religion ^b^	Muslim	1402 (32.6)
	Christian ^a^	2893 (67.4)
Baseline WHO classification ^b^	1 or 2	1355 (45.7)
	3 or 4	1608 (54.3)
Baseline CD4 count (cells/mm3) ^b^	<200	3275 (73.6)
	≥ 200	1174 (26.4)
	Median (range)	156 (0–1313)
History of Tb/HIV co-infection ^b^	No	3533 (72.1)
	Yes	1367 (27.9)
ARV adherence ^b^	Good	4064 (82.9)
	Fair or poor	836 (17.1)
Cotrimoxazole adherence ^b^	Good	4119 (94.4)
	Fair or poor	762 (15.6)
History of HIV testing ^b^	Yes	2860 (58.4)
	No	2040 (41.6)
ART shift ^b^	No	3190 (99.1)
	Yes	29 (0.9)
Baseline functional status ^b^	Work or Ambulatory	3064 (68.1)
	Bedridden	1437 (31.9)
Timing to HIV diagnosis	Early	894 (33.3)
	Late	1788 (66.7)
Clinical failure ^b^	No	2261 (80.5)
	Yes	546 (19.5)
Immunologic failure ^b^	No	3164 (80.3)
	Yes	775 (19.7)
Treatment failure ^b^	No	1493 (65.7)
	Yes	780 (34.3)

^a^ Orthodox, Catholic, Protestant

^b^ only valid percentage is calculated

### Prevalence and trend of ART discontinuation

In total, 1090 (22.3%) had discontinued, 954 (19.6%) had transferred out, 300 (6.2%) had died, 2517 (51.4%) were alive and on ART, and the remaining 39 (0.8%) had unknown outcome status in the period between 2003 and 2015 (Table 3). Of the 1090 adults who had discontinued from ART, 906(83.1%) adults had defaulted, 145(13.3%) adults had LTFU and 39(3.6%) adults had stopped during the 12 years study. The prevalence of discontinuation was consistently high between 2004 and 2007. In the period between 2008 and 2011, the proportion of discontinuation showed a different trend with a sharp decline from 6% in 2008 to 2% in 2009, a sharp rise from 2% in 2009 to 6% in 2010, and then falling in 2011 to 3%. In the recent times, the proportion of discontinuation is increasing. Table 3 shows the trend in discontinuation among HIV-infected adults on ART.

**Table 3:** Annual number of HIV infected adults on ART care and their outcomes, Southwest Ethiopia, 2003–15.

Year	New enrollment a	Death b, n(%)	Discontinuation c, n(%)	Transferred out d, n(%)	Alive & on ART e, n(%)	Total in Cohort f
2003^a^	8	0 (0)	1 (13)	0 (0)	7 (88)	8
2004	62	1 (1)	7 (10)	1 (1)	60 (87)	69
2005	468	27 (5)	51 (10)	8 (2)	442 (84)	528
2006	905	63 (5)	88 (7)	71 (5)	1125 (84)	1347
2007	574	50 (3)	148 (9)	132 (8)	1369 (81)	1699
2008	496	41 (2)	105 (6)	93 (5)	1626 (87)	1865
2009	508	39 (2)	50 (2)	103 (5)	1942 (91)	2134
2010	452	20 (1)	139 (6)	74 (3)	2161(90)	2394
2011	420	26 (1)	88 (3)	100 (4)	2367 (92)	2581
2012	352	10 (0)	96 (4)	96 (4)	2517 (93)	2719
2013	300	14 (0)	112 (4)	102 (4)	2589 (92)	2817
2014	296	7 (0)	166 (6)	147 (5)	2565 (89)	2885
2015^a^	59	2 (0)	39 (1)	27 (1)	2556 (97) ^b^	2624 ^b^
Overall		300 (6.1%)	1090 (22.3%)	954 (19.5%)	2517 (51.4%)	4900 ^b^

^a^ data from years 2003 and 2015 were not from complete number of months and were excluded from describing outcomes by a trend graph

^b^ included 39 (0.8%) patients with unknown outcome status; e = f-b-c-d; where f = e (previous year) + a (current year); b, n(%) = (b/f)*100%; c, n(%) = (c/f)*100%; d, n(%) = (d/f)*100%; e, n(%) = (e/f)*100%

### Risk factors for ART discontinuation

The results from the multiple logistic regression analysis found from the analysis of a complete case and multiple imputations (MIs) are reported in Table 4. Being female (AOR = 2.1, 95%CI: 1.7–2.8), having immunological failure (AOR = 2.3, 1.9–8.2), having Tb/HIV co-infection (AOR = 1.5, 1.1–2.1) and no previous history of HIV testing (AOR = 1.8, 1.4–2.9) were the risk factors for discontinuation.

**Table 4:** Logistic regression findings of factors affecting for discontinuation in HIV infected adults, JUTH, 2003–15.

Variable			Discontinuation status (n, %)		COR (95%CI)	AOR (95%CI): Complete cases	AOR (95%CI): Multiple imputations
			Retained	Discontinued			
Age (years)		15-<25	380 (15.1)	174 (16)	1	1	
		25-<50		2004 (79.6)	855 (78.4)	0.9 (0.8–1.1)	0.8 (0.6–1.3)	0.9 (0.7–1.1)
		50+		133 (5.3)	61 (5.6)	0.9 (0.7–1.4)	0.9 (0.5–1.1.4)	0.8 (0.5–1.2)
		Median		30	30			
**Sex**		Male	482 (44.2)	903 (35.9)	1	1	1
		Female	608 (55.8)	1614 (64.1)	1.4 (1.2–1.6) ^a^	2.1 (1.7–2.8) ^a^	1.7 (1.4–2.0) ^a^
**Marital status**		Never married	188 (25.5)	397 (25.1)	1	1	1
		Married	356 (48.2)	731 (46.2)	0.9 (0.8–1.2)		0.8 (0.4–1.6)
		Other ^a^	194 (26.3)	453 (28.7)	1.1 (0.9–1.4)		1.1 (0.9–1.3)
**Educational status**		No education	169 (23)	315 (19.6)	1		
		Primary	301 (40.9)	656 (40.8)	1.2 (0.9–1.4)	1.1 (0.6–8.3)	1.9 (0.5–5.4)
		Secondary & above	266 (36.1)	635 (39.5)	1.3(1.0–1.6)	1.8 (0.7–11.2)	1.7 (0.6–9.9)
**Religion**		Muslim	251 (34.2)	506 (31.7)	1		
		Christian ^b^	482 (65.8)	1091 (68.3)	1.1 (0.9–1.4)		
**Baseline WHO status**		Stage 1 or 2	276 (43.5)	706 (48)	1	1	1
		Stage 3 or 4	359 (56.5)	764 (52)	0.8 (0.7–1.0)	0.5 (0.2–1.8)	0.8 (0.3–2.1)
**Baseline CD4** (cells/ul)		<200	731 (78.9)	1657 (69.9)	1	1	
		> = 200	195 (21.1)	712 (30.1)	1.6 (1.3–1.9) ^a^	1.8 (0.9–2.1)	
		Median	177	129			
**Clinical failure**		No	495 (80.1)	1157 (81.4)	1		1
		Yes	123 (19.9)	265 (18.6)	0.9 (0.7–1.2)		0.8 (0.6–1.8)
**Immunologic failure**		No	726 (87.8)	1808 (77.8)	1		
		Yes	101 (12.2)	516 (22.2)	2.05 (1.6–2.6)	2.3 (1.9–8.2) ^a^	1.5 (1.3–1.9) ^a^
**HIV diagnosis**		Early	459 (33.1)	184 (34.7)	1		
		Late	927 (66.9)	347 (65.3)	0.9 (0.7–1.3)	0.8 (0.6–1.8)	
**History of Tb/HIV co-infection**		No	636 (71.7)	1305 (69)	1	1	
		Yes	251 (28.3)	587 (31)	1.1 (0.9–1.4)	1.5 (1.1–2.1) ^a^	1.4 (1.2–1.8) ^a^
**ART adherence**		Good	727 (82)	1520 (80.3)	1	1	1
		Fair or poor	160 (18)	372 (19.7)	1.1 (0.9–1.4)	1.3 (0.8–1.7)	1.6 (1.2–2.3) ^a^
**Cotrimoxazole adherence**		Good	737 (83.1)	1541 (81.8)	1		
		Fair or poor	148 (16.7)	342 (18.2)	1.1 (0.9–1.4)		
**Baseline functional status**		Working	32 (3.8)	82 (4.7)	1	1	
		Ambulatory /bedridden	801 (96.2)	1664 (95.3)	0.8 (0.5–1.2)	0.9 (0.7–2.8)	
**History of HIV testing**		Yes	643 (59)	1436 (57.1)	1		
		No	447 (41)	1081 (42.9)	1.1 (0.9–1.2)	1.8 (1.4–2.9) ^a^	1.1 (0.9–1.3)
**ART shift**		No	703 (100)	1599 (98.2)			
		Yes	0	29 (1.8)			

^a^ statistically significant at p-value ≤0.05

^b^ Orthodox, Protestant or Catholic

### Multiple imputations (MI)

We have undertaken MI to address the missing data using five imputed data sets, and we reported a model with pooled imputed values (Table 4). In estimating factors associated with discontinuation among adults, results were similar in both MI and complete case analyses except for ART adherence and history of HIV testing. ART adherence was found to be statistically significant in the MI analysis but not in the complete case analyses, and to the contrary, history of HIV testing was not found to be statistically significant in the MI analysis unlike in the complete case analyses.

## Discussion

The UNAIDS target has declared three new and ambitious goals by 2020 to end AIDS epidemic by 2030: diagnosing 90% of people living with HIV, providing ART for 90% of those diagnosed with HIV, and achieving viral suppression for 90% of patients receiving treatment [37]. Nevertheless, discontinuation from treatment challenges the success of the last two goals [38]. One in five people on ART (22.3%) had discontinued from the treatment in the current study. When compared to findings elsewhere in Ethiopia, discontinuation rate in the current study was higher than findings from the Tigray’s study [14] and lower than findings from the Amhara’s study [15]. In addition, this rate was lower than findings from studies conducted in Guinea-Bissau (51.1%)[39], Nigeria (28%)[40], and a multi-clinic study from Republic of Congo, Cameron and Burundi (83%)[41]. The dissimilarity in measurement[42], access to HIV care services[43], and innovation, adoption and implementation of cost-effective retention strategies could be the possible reasons of differences[44, 45].

For example, HIV care services such as ART accessibility affects the discontinuation rate. The estimated ART coverage[1] in Ethiopia (52%) is higher than from Guinea-Bissau (25%), Nigeria (29%), Republic of Congo (25%), Cameron (22%) and Burundi (38%). This could be one of the reasons why discontinuation rate in our finding is lower than those countries. Furthermore, the introduction of innovative programs such as Health Extension Workers (HEWs) and Health Development Army (HDA) to HIV care services[9]—programs that are not implemented in Guinea-Bissau, Nigeria, Republic of Congo, Cameron and Burundi—could affect the discontinuation rates.

The trend for discontinuation was different across years. The proportion of discontinuation in the beginning year (2004) was very high (10%) because ART was not freely available. Discontinuation had also increased from 6% in 2006 to 9% in 2007. This could be because: i) ART was universally scaled up without preparations [7]; and ii) there was inequity and limitations in access to HIV care services, a justification corroborated by 24% of the health facilities in Ethiopia provide ART care and 59% of the health facilities in the country provide prevention of mother to child transmission services[46].

The trend of discontinuation had dramatically decreased from 9% in 2007 to 2% in 2009. The overall HIV care services have been improved[6] and had a profound contribution to the reduction of discontinuation. Also, HIV was included as one of the 16 packages in a new program called health extension program (HEP)—an innovative community based health service delivery system aiming at provision of essential primary health care services—, and this program could reduce the proportion of discontinuation[47]. Nevertheless, as of 2011, the discontinuation has been growing, because non-governmental organizations has been phasing out. To the contrary, local governments have been allocating little budget for health developments, particularly for HIV/AIDS[48]. We should seriously heed the issue since the developmental assistance for health budgeted by local governments is expected to grow only slightly[49].

Hence, the magnitude of discontinuation in the current study reflects that it is a considerable number and its trend is increasing in the recent times. Programs such as community based ART distribution[9, 50], improving adherence via medication diary for care givers[51], home based nursing interventions[52] and adherence clubs[53] could increase linkage to and retention in care and further reduce discontinuation. People living with HIV should also meaningfully be involved in the continuum of care to improve retention programs[54]. The universal test, treat and keep strategy would dramatically improve ART retention and is fundamental in cost-effective HIV care[55].

Compared to males, females were more likely to discontinue from ART, a similar finding to previous studies [56, 57]. Part of the reasons for this could be due to: (i) HIV related stigma which is known to be higher in females than in males, and prohibits them out from HIV care seeking [58, 59]; (ii) Lower literacy status in females than males, which is a big challenge that prevent women from optimising the benefit of HIV care [60], (iii) higher usage in females than males of traditional healers which hinders females from taking ART consistently [61], and (v) lack integration between modern and traditional medicines which has been identified as one of the key factors in for a consistent uptake of ART programs in Ethiopia [62]. Similar to findings by Meloni and colleagues[63], discontinuation was higher among patients with immunological failure than their comparator. HIV-infected patients with immunological failure are susceptible to multiple opportunistic infections and do progress to the advanced stage of HIV/AIDS rapidly leading to quick deterioration of their health status[64, 65]. Poor health status can also be among significant factors that deter them from uptaking HIV care services consistently [66]. To address ART discontinuation problem, Programs such as linkage-case-management (LCM)[67] that focus on multiple points of HIV care to enhance retention have been suggested as a good practice.

Although it has been stated that Tb/HIV co-infection mortality and morbidity was reduced dramatically in the era of ART [68, 69], in the current study, the odds of discontinuation was higher in Tb/HIV con-infected patients than in patients with HIV infections alone. These results are consistent with findings from earlier studies [70–72]. The intimate linkage between HIV and Tb enables the progression of HIV disease to advanced stage rapidly and thereby disallowing patients from regular treatment intake [26, 73]. In addition, the double stigma related to Tb/HIV coinfection as well as the double burden of having to take multiple pills for both conditions (pill effect) could be a compounding factor for discontinuation within this cohort. Because the burden of Tb/HIV confection is a serious problem in Ethiopia and in similar settings, it is vital to implement intervention strategies that strengthen access to universal Tb/HIV co-infection care throughout the country. It is necessary for the special attention to be given to Tb/HIV co-infection because it is well known that Tb causes the highest mortality among HIV-infected patients[38]. The lack of history of HIV testing in association with discontinuation could be linked with poor awareness of the care[7, 74], high HIV-related stigma[59], and feeling of wellbeing[75]. Although the outcomes status of current study was not known, previous studies in Ethiopia and elsewhere have shown that 9% of transferred out and 40–86% of LTFU cases failed to re-engage to the care[76], and half of LTFU patients after tracing were found deceased [7, 23].

It is necessary to acknowledge limitations of the current study including: i) some variables such as stigma and mental illness were not assessed due to the nature of the study design—previous studies reported that stigma[77] and mental illness[24] affect ART discontinuation; ii) the outcome status of discontinued patients was not known, iii) findings of this study might not infer the other level of health facilities such as health centers or private hospitals; and iv) the comparison for discontinuation may not be appropriate due the dissimilarity in definition between studies.

## Conclusions

The magnitude of discontinuation was recorded among one in five patients, and the trend has increased recently. Discontinued patients were more likely to be females, Tb/HIV co-infected, with immunological failure and no history of HIV testing. Additionally, in order to ensure the best outcomes and enhance the course of HIV care it is necessary that patients should be tracked to establish whether they have defaulted, transferred out, or died. We recommended implementing community ART distributions, strengthening adherence clubs, and adopting benchmarking programs such as linkage-case-management to enhance ART linkage and retention which have demonstrated to be effective in similar settings. Ethiopia has to subscribe into the East African International epidemiologic Databases to Evaluate AIDS (EA-IeDEA) Consortium[78], not only to share uniform measurements and gain research networks, but learn good practices in implementing HIV/AIDS treatment and overall care and management.

## Abbreviations

AIDS: acquired immunodeficiency syndrome
ART: antiretroviral therapy
CD4: cluster of differentiation 4
MIs: multiple imputations
JUTH: Jimma University Teaching Hospital
HIV: Human Immunodeficiency virus
SSA: Sub-Saharan Africa
Tb: tuberculosis
UNAIDS: The Joint United Nations Program on HIV and AIDS
WHO: World Health Organization
